# Evaluation of the Effectiveness of Probiotic Mouthwashes in Reducing Dental Plaque in Primary and Permanent Teeth: A Randomized Clinical Trial

**DOI:** 10.7759/cureus.28125

**Published:** 2022-08-18

**Authors:** Enas Alhallak, Chaza Kouchaje, Ahmad Hasan, Ramah Makieh

**Affiliations:** 1 Pediatric Dentistry, Faculty of Dental Medicine, Damascus University, Damascus, SYR; 2 Pharmaceutical Chemistry and Quality Control of Medicament, Faculty of Pharmacy, Damascus University, Damascus, SYR

**Keywords:** orphan children, dental plaque, mouthwashes, fluoride, probiotic

## Abstract

Background: Due to the disadvantages of chemical mouthwashes, the search for an effective and safe anti-plaque agent has led to the emergence of probiotics.

Aim: To compare the effectiveness of nonchemical mouthwashes (probiotic) with chemical mouthwashes (fluoride) on plaque accumulation in orphan children after seven, 14, and 30 days of use.

Materials and methods: The present study was a triple-blind randomized controlled trial with two parallel groups (A and B), which included 30 healthy children. Each group included 15 children aged between eight and 10 years from Dar Al-Rahma Orphanage in Damascus, Syrian Arab Republic. Group A used 10 ml of probiotic mouthwash (ProbioClean) and group B used 10 ml of fluoride mouthwash (Colgate) for 60 seconds for 30 days. Turesky Modified Quigley-Hein (TMQH) plaque index was used in this study to record the values of plaque accumulation on days seven, 14, and 30.

Results: Mann-Whitney U test showed statistical significance between probiotics and fluoride mouthwashes on days 14 (p < 0.001) and 30 (p = 0.001), and there was no statistical significance on day seven (p = 0.934).

Conclusion: According to the results of this study, probiotic mouthwashes are considered an effective solution for maintaining oral health. However, probiotics are more effective in reducing plaque accumulation after a month of use.

## Introduction

Dental caries is the most common health problem, which affects 60-90% of children and adolescents. The state of caries differs throughout the world, for example, low-income countries have more caries with more sugar exhaustion [1], especially in orphan children who have more dental decay and worse oral health. Generally, orphan children only receive dental care in emergency cases [2]. Dental caries is caused by a specific and highly variable microbial community called dental plaque [3]. Many techniques were used to control plaque accumulation such as tooth brushing, dental flossing, and mouthwashes [4]. Research studies have shown that mechanical plaque control is insufficient, and because of that, mouthwashes may help in controlling dental plaque and dental caries. In addition, it is a preferred method for patients because it is easy and does not require any skill [5]. Mouthwash is a safe and effective method for the prevention of bacterial growth and reducing permanent colonization by the delivery of antimicrobial agents. These agents can prevent bacterial adhesion, colonization, and metabolism [6,7]. However, the daily use of these agents has many side effects like teeth coloration and drug resistance. To avoid these disadvantages, probiotic therapy can be considered an available alternative to mouthwashes [8]. The World Health Organization has defined probiotics as “live microorganisms which, when administered in adequate amounts, confer a health benefit on the host.” Hence, the present study aims to compare the effectiveness of nonchemical mouthwashes (probiotics) with chemical mouthwashes (fluoride) on plaque accumulation in orphan children after seven, 14, and 30 days of use.

## Materials and methods

The present study was a randomized controlled trial that included 30 healthy children (12 males and 18 females) assigned into two parallel groups (A, B). Each group consisted of 15 children aged between eight and 10 years from Dar Al-Rahma Orphanage in Damascus, Syrian Arab Republic. Ethical approval for this study was obtained from the Institutional Ethical Committee of the Faculty of Dentistry, Damascus University, Syria (IRP NO.UDDS-336-2782018/SRC1450).

The healthy children with good oral health who recorded in dental examination the existence of permanent incisors and first permanent molars and no decay on the buccal surface of examined teeth were included in this study. However, children with physical and mental disabilities, sensitivity to one of the research materials, children who took antibiotics, anti-inflammatories, and oral rinses for at least four weeks before this study, or who underwent a preventive program during the past three months before the start of this study were excluded.

Randomization

Random sampling was employed by the random number table method and every group randomly used one mouthwash by lottery method. The random allocation sequence was created by one of the authors using the random number table method. The random allocation sequence was hidden from the main investigator until mouthwashes were appropriated to the individuals. The major investigator registered the study subjects and evaluated the study index.

Blinding

The blinding was controlled by a third person (dentist) who divided mouthwashes into simple plastic bottles of the same specific size for group A and group B. All participants and the researcher did not know the contents of the bottles. The third person detected the contents after the end of the study. The statistician was also blinded; therefore, this was a triple-blind study.

Methods

All children were taught to brush their teeth by circular method before a week of starting the study, and it was confirmed that they mastered the method. Identical new brushes and non-fluoride toothpaste were distributed to all of them. In addition, scaling and polishing were done before starting the study. When the study started, group A used 10 ml of probiotic mouthwashes (ProbioClean), and group B used 10 ml of fluoride mouthwashes (Colgate) for 60 seconds. The use of mouthwashes by all the children was monitored by the main investigator.

**Figure 1:**
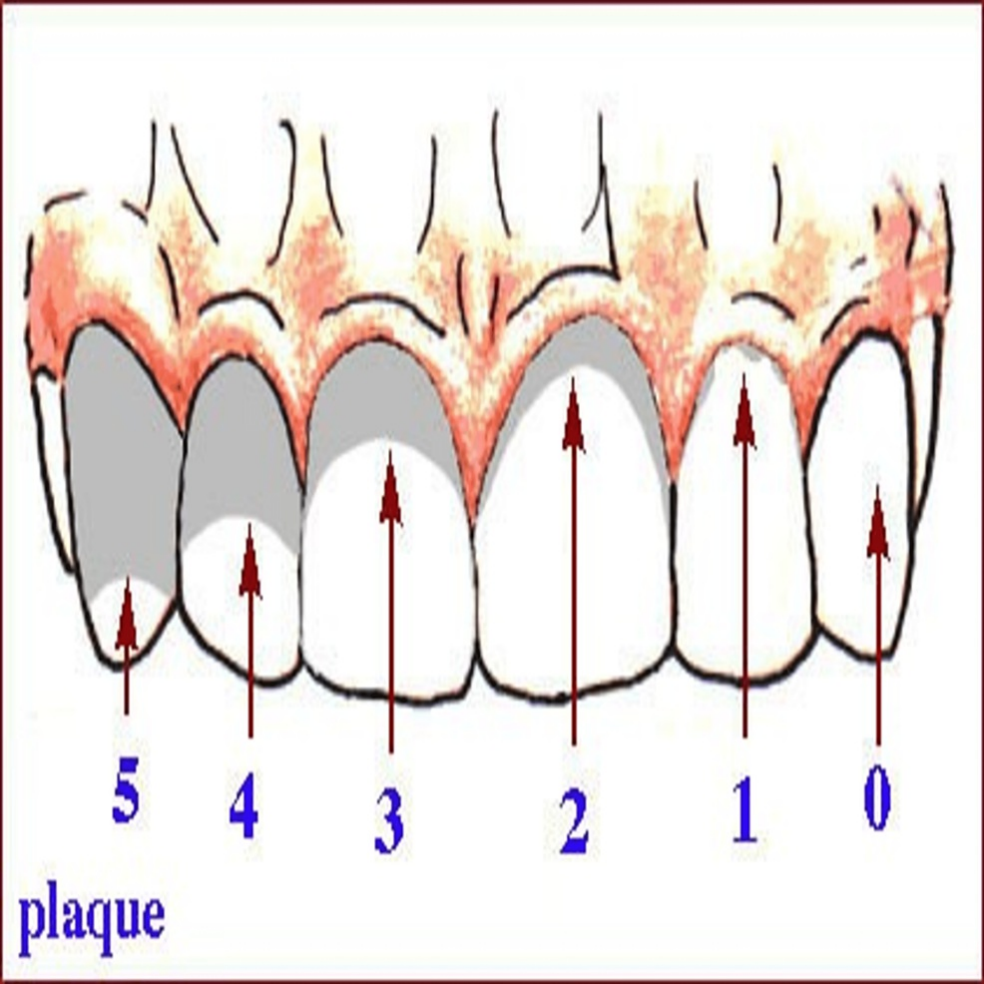
Quigley-Hein plaque index Adapted from: Chinger G, Hadidjah D, Rusminah N. (2012). Comparison effectiveness between cetylpyridinium chloride and triclosan mouthwash on plaque. Padjadjaran Journal of Dentistry, 24(3). doi: http://dx.doi.org/10.24198/pjd.vol24no3.26838. Under Creative Commons Attribution 4.0 International License.

Mira-2-Ton (Hager & Werken, Duisburg, Germany) plaque disclosing solution was applied on the buccal surface of permanent incisors, first permanent molars, and primary second molars in both maxillary and mandibular jaw. Turesky Modified Quigley-Hein (TMQH) plaque index was used in this study to record the values of plaque accumulation on days seven, 14, and 30, as seen in Figure 1 and Table 1.

**Table 1:** Turesky Modified Quigley-Hein (TMQH) plaque index scoring criteria (1970)

Score	Description
0	No plaque
1	Isolated areas of plaque at the gingival margin
2	A thin band of plaque at the gingival margin (≤1 mm)
3	Plaque covering up to 1/3 of tooth surface
4	Plaque covering 1/3 to 2/3 of tooth surface
5	Plaque covering ≥ 2/3 of tooth surface

Statistical analysis

The normality of the data was checked using the Kolmogorov-Smirnov analysis, and the result revealed a non-normal distribution. Mann-Whitney U tests were used to study the difference in plaque scores between the groups of teeth. In this study, the level of significance (p-value) was set at 0.05, and all statistical analyses, including descriptive analysis, were done using SPSS version 26 (IBM Corp., Armonk, NY).

## Results

The plaque accumulation was calculated after seven, 14, and 30 days of using mouthwashes. The comparison between the anterior and posterior teeth is given in Table 2, and between the primary and permanent teeth is given in Table 3. The mean of the whole mouth is given in Table 4.

**Table 2:** Plaque accumulation comparison between the anterior and posterior teeth on days seven, 14, and 30 Mann-Whitney U test. Significance at p < 0.05.

	Probiotic (mean rank)			Fluoride (mean rank)		
	Permanent first molars	Permanent anterior teeth	P-value	Permanent first molars	Permanent anterior teeth	P-value
Day 7	104.3	73.1	0.002	109.5	70.6	0.000
Day 14	125.5	49	0.000	111.8	70.2	0.000
Day 30	132	53.3	0.000	125.3	52.3	0.000

**Table 3:** Plaque accumulation comparison between primary and permanent teeth on days seven, 14, and 30 Mann-Whitney U test. Significance at p < 0.05.

	Probiotic (mean rank)			Fluoride (mean rank)		
	Permanent first molars	Primary second molars	P-value	Permanent first molars	Primary second molars	P-value
Day 7	104.3	94.1	0.795	109.5	91.4	0.126
Day 14	125.5	94	0.000	111.8	89.5	0.043
Day 30	132	86.2	0.000	125.3	94	0.002

**Table 4:** Plaque accumulation of the whole mouth Mann-Whitney U test. Significance at p < 0.05.

	Probiotic (mean rank)	Fluoride (mean rank)	P-value
Day 7	3.80	3.99	0.934
Day 14	2.44	3.62	0.000
Day 30	2.21	2.83	0.001

Mann-Whitney U test showed statistical significance between probiotic and fluoride mouth rinses on days 14 (p < 0.001) and 30 (p = 0.001), and there was no statistical significance on day seven (p = 0.934).

In comparison between the anterior and posterior teeth, there was a statistical significance in the probiotic group and fluoride group. The plaque accumulation in anterior teeth was less than in posterior teeth on days seven, 14, and 30.

In comparison between the primary and permanent teeth, there was no statistical significance in the probiotic group (p = 0.795) and fluoride group (p = 0.126) on day seven, but on day 14 and day 30, there was a statistical significance in both groups.

## Discussion

Health starts from the mouth, and medical scientific research shows this all the time. Improving oral health can improve the quality of life of individuals and society [9], especially in a low-income country that has low fluoride exposure (in the water supply and oral health products, for example, toothpaste) and poor access to oral healthcare services in the community [10]. One of the high-risk groups of poor oral hygiene are orphans who have more dental caries because of the lack of dental knowledge in the orphanage [2]. Mechanical oral health products such as toothpaste, dental floss, and intra-proximal brushes can play a major role in supra-gingival plaque removing and controlling dental caries and periodontal disease [11,12]. Several studies have shown that plaque is not completely removed using mechanical methods alone, as many patients are untrained or unable to use it effectively.

Therefore, chemical control methods appeared as an aid and enhancer technique [13]. Oral washes are used as a support for mechanical dental cleaning methods to reduce dental plaque [12]. The American Dental Academy introduced fluoride mouthwashes as a therapeutic product for preventing dental decay by fluoride ions, which promote remineralization and inhibit bacterial growth and metabolism [14]. However, the illogical use of fluoride has dangerous side effects like dental fluorosis, natural fluorosis, neurotoxicity, and others [15]. To avoid this disadvantage, probiotic technology was presented by Elie Metchnikoff in 1908 [16]. Probiotic mouthwashes contain living microbes such as lactobacilli or *Bifidobacterium*, which are considered a part of oral microflora and could reduce the level of *Streptococcus mutans* in saliva by several mechanisms such as the production of antimicrobial agents (lactic acid, hydrogen peroxide, and bacteriocins), modulating the inflammatory response, and competing with pathogens for adhesion surfaces [17].

This study compared the efficacy of probiotic and fluoride mouthwashes on plaque accumulation in three sections of the mouth: permanent anterior teeth, primary second molars, and permanent first molars. Permanent first molars are the most sensitive teeth to caries in young people aged eight to 10 years [15]. The results obtained showed that there was no significant difference between the groups after seven days of use, but after 14 and 30 days, the probiotic group showed more effectiveness in decreasing plaque accumulation than the fluoride group, which could be due to the drug resistance of *S. mutans*. Breaker studied the effects of fluoride on oral bacteria and proposed that fluoride riboswitch of *S. mutans* has the ability to push the fluoride ion from the cell membrane, and the resistance develops in that way [18]. Jothika et al. studied the colony counts of *S. mutans* after 30 days of using probiotic mouthwashes and proved the decrease in the bacterial count was sustained after the 30th day of usage of the probiotic mouthwash [19]. In a comparison of plaque accumulation between anterior and posterior teeth, anterior teeth had less degree of plaque in the three days in both groups and this agrees with Sreenivasan et al.'s study who studied plaque index in the dental arch and proved that anterior surfaces had lower plaque degree than posterior surfaces [20].

In a comparison between primary and permanent molars, after seven days, there was no difference, which agrees with Ramberg et al.’s study who proved that plaque formed during the seven days of the experiment was similar in the primary and permanent teeth [21]. After 14 and 30 days, plaque accumulation was statistically significant, and primary molars had a lower degree than permanent molars, which could be due to the difference in the anatomy of the teeth.

Limitation

A limitation of the study is the use of mouthwashes only one time daily because of the policy of the orphanage.

## Conclusions

According to the results of this study, it can be concluded that probiotic and fluoride mouthwashes are considered effective solutions for maintaining oral health; however, probiotics are more effective than fluoride in reducing plaque accumulation after a month of use. The authors suggest the promotion of probiotic mouthwash after conducting clinical trials on a larger scale, so that risk of adverse effects is reduced and general health is promoted along with oral health.
